# Prevalence of malaria and dengue co-infections among febrile patients during dengue transmission season in Kassala, eastern Sudan

**DOI:** 10.1371/journal.pntd.0011660

**Published:** 2023-10-04

**Authors:** Khider Alsedig, Mawahib H. Eldigail, Adel Hussein Elduma, Arwa Elaagip, Omnia Altahir, Hanaa Adli Siam, Yousif Ali, Tajeldin Abdallah

**Affiliations:** 1 Department of Medical Entomology, National Public Health Laboratory, Federal Ministry of Health, Khartoum, Sudan; 2 Epidemiology Department, National Public Health Laboratory, Federal Ministry of Health, Khartoum, Sudan; 3 Department of Parasitology and Medical Entomology, Faculty of Medical Laboratory Sciences, University of Khartoum, Khartoum, Sudan; 4 Department of Epidemiology, Tropical Medicine Research Institute, National Center for Research, Khartoum, Sudan; 5 Health Emergencies and Epidemics Control General Directorate, Federal Ministry of Health, Khartoum, Sudan; 6 Department of Internal Medicine and Microbiology, Faculty of Medicine, University of Kassala, Kassala, Sudan; Australian Red Cross Lifelood, AUSTRALIA

## Abstract

**Background:**

Malaria and dengue are common mosquito-borne diseases around the world that cause high mortality and morbidity. The number of cases of both diseases is currently rising in Sudan and is associated with climate and environmental changes. Limited information is available on malaria and dengue co-infections and the severity of the two diseases among febrile patients in eastern Sudan. Thus, this study aimed to estimate the prevalence of malaria and dengue co-infections among febrile patients in Kassala, eastern Sudan.

**Methodology/Principal findings:**

A cross-sectional hospital-based study was conducted among febrile patients from September to December 2019. A total of 395 patients were enrolled after consenting to participate in the study. Demographic and clinical data were collected by structured questionnaires. Blood samples were provided to diagnose malaria infections using microscopy and polymerase chain reaction (PCR) and for serology diagnosis of dengue using enzyme-linked immune sorbent assay (ELISA) IgM. Multiple logistic regression analysis was used to assess the association between demographic information, clinical symptoms and malaria and dengue co-infections.

Out of 395 febrile patients examined 158 (40%) were malaria positive and 67 (17%) were dengue positive. The prevalence of malaria and dengue co-infections was 6.6% (26/395). Results of multiple logistic regression indicated that elder patients (41–60 years) had less rate of co-infections (OR = 0.3, 95% CI 0.11 to 0.81, *p*-value = 0.018), while patients of co-infections were eight times more likely to have fatigue, and two times more likely to suffer from joint and muscle pain and this difference was statistically significant with (OR = 8.3, 95% CI: 1.89 to 37.22, *p*-value = 0.005) and (OR = 2.4, 95% CI 1.10 to 5.39, *p*-value = 0.027), respectively.

**Conclusions/Significance:**

This study confirmed the existence of malaria and dengue co-infections among febrile patients in Kassala, eastern Sudan for the first time. The severity of clinical symptoms of patients with malaria and dengue co-infections was observed, and the co-infections were found prevalent among young people.

## Author summary

Malaria and dengue are common vector-borne diseases in eastern Sudan. However, co-infections with these two diseases are not reported among febrile patients. Malaria and dengue co-infections need a particular concern because this may increase morbidity and mortality if there are delays in diagnosis and appropriate treatment. So, this study has investigated the prevalence of malaria and dengue co-infections among febrile patients in Kassala, eastern Sudan. The findings of the study showed the prevalence of malaria and dengue co-infections in the study area according to the gender and age of enrolled patients. Results also showed the severity of clinical symptoms of patients with malaria and dengue co-infections were high compared to mono-infections, and the co-infections were found less among elder people.

## Introduction

Both malaria and dengue are known to be rapidly spreading mosquito-borne diseases and are of high importance in terms of morbidity and mortality, posing a worldwide public health problem due to the global expansion of their vectors [1]. Malaria is a protozoan parasitic infection caused by apicomplexan *Plasmodium* spp. which is usually transmitted by *Anopheles* spp. mosquitoes. Five parasite species cause malaria in humans; nevertheless, two of these species–*P*. *falciparum* and *P*. *vivax*–pose the greatest threat. *Plasmodium falciparum* is the deadliest malaria parasite and the most prevalent on the African continent [2]. Dengue infection is caused by any of the four distinct serotypes (DEN-1, 2, 3, and 4) of single-stranded RNA flavivirus, and is mainly transmitted by *Aedes aegypti* mosquitoes [3]. Dengue is considered the most important arbovirus in terms of morbidity and mortality, putting almost half of the population at risk of infection in the world. Fever and other symptoms rarely last more than seven days, but convalescence can be prolonged and debilitating. Although people can obtain immunity to one of the dengue serotypes, they are still susceptible to the others. The disease is now endemic in more than 100 countries worldwide. The incidence of this disease has increased dramatically, especially the incidence of the more severe dengue hemorrhagic fever (DHF) [4].

Malaria and dengue are endemic in similar tropical regions, and therefore, may result in the possibility of co-infection. Urban demographic expansion, deforestation, and agricultural settlements in peri-urban areas are known causes of the probability increasing of concurrent infections of these two diseases [5]. Concurrent infections of malaria and dengue are due when both diseases co-occur in an individual. Since there are similarities in the clinical characteristics between these two infections, the diagnosis of malaria and dengue co-infections might be either misdiagnosed or misinterpreted as mono-infection. However, it is important to differentiate between the two infections, as this may increase morbidity and mortality if there are delays in diagnosis and appropriate treatment.

There are many cases of malaria and dengue co-infections reported from various regions in the world following the first case that was reported in France in July 2005 [6]. Several studies have been published reporting the co-circulation of malaria and dengue [7,8]. The reported frequencies of concurrent infections in febrile patients presenting to outpatient departments of hospitals in various studies have been quite variable and range from 1% in French Guiana to 6% in India, and 27% in Pakistan [9]. Apart from shared endemicity, the two diseases also share similar clinical presentations with febrility as the most common symptom [10].

Malaria is endemic in Sudan, whereas dengue has established itself in an epidemic transmission cycle [11]. The major outbreaks of dengue in Sudan were caused by serotypes 1, 2, and 3 that circulated in different parts of Sudan [12,13]. Over 80%, 58%, and 33% of the cases tested for dengue in West, Central, and East Darfur, respectively, also tested positive for malaria, according to an analysis of the Sudanese records keeping track of health facilities. This interesting observation points out the high level of co-infections with malaria amongst Sudanese patients with severe symptoms who tested positive for dengue fever and has implications for vector control approach and disease surveillance activities [14]. Kassala witnessed increased cases of dengue during the last three years according to the local clinical services records [15]. Malaria has become a major health problem in the last few years with an incidence rate of 140 cases per 10,000 population in Kassala state [16]. Clinical manifestations include symptoms that are non-specific and cannot be distinguished from other febrile illnesses. In addition, the misdiagnosis of both malaria and dengue infections may also lead to ambiguity in terms of disease burden in this area. Conducting a laboratory survey for malaria and dengue co-infections in Kassala city is necessary to assess the burden of concurrent infections and will reveal the potential possibility of existing co-infections. Therefore, this study was conducted to assess the burden and the risk factors associated with malaria and dengue co-infections in Kassala, eastern Sudan.

## Methods

### Ethics statement

Ethical approval for the study was provided and issued by the Federal Ministry of Health, Sudan (No. 4-11-18). A signed informed consent was obtained from each participant that agreed to participate voluntarily in this study. All personally identifiable information was not disclosed to any person except the main investigators. The cost of ELISA and PCR tests for patients was paid by the authors and the participants had not any payment for these tests.

### Study site

**Fig 1:**
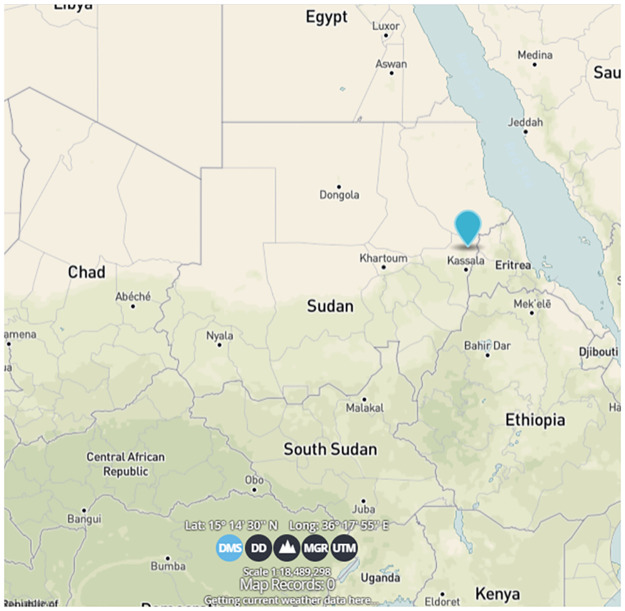
Local map of the study sites in Kassala state, eastern Sudan. The map was used from the public domain of U.S. Geological Survey (USGS) (http://www.usgs.gov), the link of the map (https://ngmdb.usgs.gov/topoview/viewer/#5/13.774/28.015).

Kassala state is located in eastern Sudan (10°12′N 34°19′E) (Fig 1). It is bordered by Eritrea and Ethiopia. A seasonal river “Ghash River” crosses Kassala city (the capital) longitudinally, and divides it into eastern and western regions. The land space of Kassala city is 1.115 km^2^. Kassala city is located along the main Khartoum-Port Sudan highway which makes it the main trading center beside the connections with Eritrea and Ethiopia. The population of Kassala is estimated about 401,477 [17]. In the northern parts of the state, the climate is the Red Sea climate, while in the other parts; the environment is desert, semi-desert, valley, and savannah climate with large fruit farms inside the Kassala locality. The average rainfall is 350 to 911 ml and the average temperature is 30°C to 51°C.

### Study design and sampling technique

A cross-sectional hospital-based survey for malaria and dengue co-infections was conducted in Kassala city. The study participants were enrolled during the dengue transmission season between September to December 2019 from eight hospitals and health centers namely Kassala Teaching Hospital, Rama Specialized Hospital, Alsika Hadid Health Center, Kassala Kuwaiti Hospital, Kassala Police Hospital, Doctors Health Center, Tarawa Health Center, and Alswagi Algnobia Health Center in Kassala city.

### Study population

In this survey, all febrile patients who were residents and admitted to the hospitals and health centers in Kassala city and agreed to participate in the study and signed the informed consent were enrolled in the study. Study participants were examined for malaria infection using microscopy and positive cases were confirmed using the polymerase chain reaction (PCR) method, patients were also examined for dengue infection using the serology method of enzyme-linked immune sorbent assay (ELISA IgM) [18].

### Data collection

A well-constructed questionnaire was used to collect data from febrile patients. Demographic variables such as sex, age, and residence were included in the questionnaire. Also, clinical symptoms like fever, headache, fatigue, and nausea were collected from study participants. Data were collected through face-to-face interviews using a paper-based data collection tool.

### Outcome variables

The first outcome variable was malaria and dengue co-infections whether it was *P*. *falciparum* or *P*. *vivax*. The second outcome was the co-infections between dengue and *P*. *falciparum*. The third outcome variable was *P*. *vivax* and dengue co-infections. Each outcome variable had a binary outcome, either positive or negative.

### Laboratory diagnosis

A training workshop for laboratory technicians and health workers was conducted before the beginning of the study to review the standard procedures for malaria and dengue examinations and diagnostics. Venous blood (about 3 ml) was collected from eligible participants in the ethylenediaminetetraacetic acid (EDTA) containers. A few drops were used for the preparation of thin and thick smears that were stained with Giemsa stain and used for the detection of malaria parasites. All the positive microscopy samples were confirmed using the molecular technique. About 400 μl of blood from each positive microscopy sample was used for DNA extraction using the guanidine chloride protocol [19]. Then, outer and nested PCR was performed to detect genes of the malaria parasite following Snounou and Singh method [20].

The PCR products were separated in 1.5% agarose gel stained with ethidium bromide and observed under UV using the BioDocAnalyze gel image documentation system (Biometra Analytika Jena Company, Germany).

### ELISA IgM analysis

The IgM antibodies specific for dengue virus NS1 serotypes (1, 2, 3, and 4) were detected using an anti-dengue virus ELISA (IgM) kit (Euroimmun, Germany) [21] following the manufacturer instructions. The IgM antibodies appeared on the 4^th^ day of dengue virus infection and the highest IgM mean titers were observed on day 7^th^ of infection. The IgM antibodies were not detected after the 60^th^ day of dengue infection [18].

### Data management and statistical analysis

All demographic, clinical, and laboratory data were entered into the excel sheet and stored in a computer until the time of analysis. Each participant was assigned a unique identifier so that identifying information (names and residential address) was removed upon entering the questionnaire data into the database and confidentiality was maintained. Descriptive statistics were performed based on variables from the questionnaire using STATA version 14. Measures of central location such as mean, median, and measures of dispersion such as standard deviations, and interquartile range were calculated for continuous variables in the questionnaire. Proportions and ratios were calculated for categorical variables such as sex, age group, and others. Measure of statistical significance such as *Chi-square* analysis was used to test the significant associations between outcome variables and explanatory variables. Multiple logistic regression was used to estimate the odds ratio (OR) and 95% confidence interval (CI) for the outcome variables. For all the analyses, variables were considered statistically significant at *p-*value of ≤0.05.

## Results

A total of 395 febrile patients were included in this study with a median age of 26 years (range: 5–84). Male patients counted for 229 (58%) and females for 166 (42%). The majority of patients (n = 160, 40%) were found in the age group of 21–40 years (S1 File).

Out of 395 febrile patients examined 158 (40%) were malaria positive and 67 (17%) were dengue positive. The total of malaria and dengue co-infections was 26 (6.6%). Detailed results of malaria species infections with or without dengue co-infections were presented in (Table 1). All the eight hospitals included in the study reported malaria infections, six of them reported dengue cases and only four hospitals reported malaria and dengue co-infections (Table 2). Regarding the clinical symptoms, out of 395 patients, 357 (90.4%) suffered from headache, 302 (76.5%) from fatigue, 209 (52.9%) from nausea, 163 (41.3%) from vomiting, and 253 (64.1%) from joint and muscle pain.

**Table 1:** Distribution of malaria and dengue infections among febrile patients in Kassala, eastern Sudan.

Cases	Number	Percentage %
Positive for *Plasmodium falciparum*	111	28.17
Positive for *P*. *vivax*	47	11.93
Positive for dengue IgM	67	17.01
Positive for *P*. *falciparum* and dengue	12	3.04
Positive for *P*. *vivax* and dengue	15	3.8

**Table 2:** Detailed information about the enrolled participants from different hospitals and health centers in Kassala city, eastern Sudan showing their infection by malaria, dengue, and malaria and dengue co-infections.

Name of health facility	Positive for malaria	Positive for dengue	Positive for malaria and dengue	Total of enrolled participants
Kassala Teaching Hospital	46 (37.7%)	14 (11.5%)	5 (4.1%)	122
Rama Specialized Hospital	30 (37.0%)	23 (28.4%)	10 (12.3%)	81
Alsika Hadid Health Center	27 (40.9%)	21 (31.8%)	9 (13.6%)	66
Kassala Kuwaiti Hospital	0 (0%)	0 (0%)	0 (0%)	46
Kassala Police Hospital	38 (95%)	2 (5%)	2 (5%)	40
Doctors Health Center	13 (81.3%)	0 (0%)	0 (0%)	16
Tarawa Health Center	3 (25%)	2 (16.7%)	0 (0%)	12
Alswagi algnobia Health Center	1 (8.3%)	2 (16.7%)	0 (0%)	12

According to gender, the results of malaria and dengue co-infections were reported in (Table 3) and S2 File. According to the age groups, only two age groups (5–20) and (21–40) years were infected with *P*. *falciparum* and dengue co-infections (4/134, 2.9%) and (8/160, 5.0%) respectively (Table 3 and S1 File).

**Table 3:** Clinical symptoms of different malaria species and dengue co-infections in Kassala city, eastern Sudan.

Parameter	DENV/*P*.*falciparum* N/total (%)	*p*-value	DENV/*P*.*vivax* N/total (%)	*p*-value
**Age groups**				
5–20	4/134 (2.9)	1.00	5/134 (3.7)	0.162
21–40	8/160 (5.0)	0.521	7/160 (4.4)	0.047
41–60	0	0.004	2/82 (2.4)	0.271
61+	0	0.038	1/18 (5.5)	0.006
**Sex**				
Female	7/166 (4.21)	0.821	6/166 (3.61)	0.244
Male	5/229 (2.18)	0.019	9/229 (3.9)	0.008
**Clinical symptoms**				
Fever	15/387 (3.9)	0.008	12/387 (3.1)	0.052
Headache	12/357 (3.4)	0.091	15/357 (4.2)	0.031
Fatigue	12/302 (4.0)	0.114	14/302 (4.6)	0.112
Nausea	9/209 (4.3)	0.442	10/209 (4.8)	0.083
Vomiting	7/163 (4.3)	0.665	5/163 (3.1)	0.771
Bleeding	0	0	1/8 (12.5)	0.006
Joint and muscle pain	8/253 (3.2)	0.145	15/253 (5.9)	0.003

The results of multiple logistic regression analysis showed that patients in the age group (41–60) had less rate of *P*. *vivax* and dengue co-infections (OR = 0.3, 95% CI: 0.11–0.81, *p*-value = 0.018). In addition, patients infected with *P*. *vivax* and dengue co-infections are eight times more likely to have fatigue compared to those who did get the mono-infection (OR = 8.3, 95% CI: 1.89–37.22, *p*-value = 0.005). Also, patients with *P*. *vivax* and dengue co-infections are two times more likely to suffer from joint and muscle pain and this difference was statistically significant (OR = 2.4, 95% CI: 1.10–5.39, *p*-value = 0.027). For the *P*. *falciparum* and dengue co-infections, no statistical significant association was observed (Table 4).

**Table 4:** Results of multiple logistic regression analysis of the factors associated with *P*. *vivax* and dengue co-infections in Kassala state, eastern Sudan.

Variable	OR (95% CI)	*p*-value
**Sex** (Male)	0.7 (0.40, 1.47)	0.437
**Age groups**		
5–20	1	
21–40	0.5 (0.25, 1.08)	0.083
41–60	0.3 (0.11, 0.81)	0.018
61+	0.4 (0.93, 2.18)	0.322
**Clinical symptoms**		
Fatigue (Yes)	8.3 (1.89, 37.22)	0.005
Nausea (Yes)	0.9 (0.46, 2.09)	0.974
Vomiting (Yes)	1.4 (0.68, 2.89)	0.359
Joint and muscle pain (Yes)	2.4 (1.10, 5.39)	0.027

## Discussion

Malaria and dengue co-infections are considered of great public health importance, especially in the tropics. This condition is usually underreported and could have fatal outcomes if left undiagnosed. However, malaria and dengue co-infections cause febrile illness with similar symptoms that are not easy to differentiate clinically. Therefore, understanding the distribution of malaria and dengue co-infections is vital for improving accurate diagnosis and proper therapeutic interventions. Information provided by this study can be used as guidance for clinicians, policymakers, and public health workers for choosing appropriate diagnostics and treatment in Sudan and similar endemic areas.

Many areas in Sudan reported high prevalence of both malaria and dengue infections such as in the eastern region, but there were no previous reports about co-infections from this region. In the current study, the prevalence of malaria and dengue co-infections was reported (n = 26/395, 6.6%) among febrile patients for the first time in Kassala, eastern Sudan. This finding is lower than a study conducted in western Sudan where the prevalence of malaria and dengue co-infections was 15% [22].

The finding of previous studies conducted in endemic areas of dengue fever and malaria found a high prevalence of co-infections such as a study conducted in Brazil in 2009, where 11 co-infections were recorded among 132 patients with *vivax* malaria showing a prevalence of 8.3% [23]. In another study during a dengue outbreak in India, the prevalence of co-infections was estimated as 5.8% among all cases of fever (77 out of 546) [24]. Additionally, the highest prevalence was estimated in Pakistan as 23.2% [8]. On the other hand, our finding was relatively less than a study conducted in French Guiana, where the prevalence of malaria and dengue co-infections was 7.1% (17 of 238) [25]. The difference in the prevalence of malaria and dengue co-infections may be attributed to many factors, like local endemicity and dissimilarity in socio-demographic characteristics. Malaria and dengue diseases are sharing similar clinical symptoms, particularly at the initial phase of the disease and can presented with febrile illness symptoms [1]. So, it is difficult to identify malaria and dengue co-infections due to the difficulty in distinguishing their similar clinical symptoms. In resource-limited settings particularly during outbreaks, clinicians might not have the resources or the time to do detailed investigations [26]. This might lead to delayed diagnosis of the malaria and dengue co-infections which may cause serious prognoses for patients. A study conducted in East Timor reported that due to delayed diagnosis of malaria and dengue co-infections, the patient was died [27].

Based on our findings, it was observed that malaria and dengue co-infections were found not to be associated with age groups, but less associated among elder age group (41–60) years. Also, malaria and dengue co-infections were not associated with gender. This finding is in agreement with a study conducted in Cameroon where gender and age were not associated with malaria and dengue co-infections [28]. Contrary to our finding, a study conducted in Cameroon reported that age group of (30–45) years was associated with malaria and dengue co-infections [29].

It was observed that malaria and dengue co-infections increase the likelihood of suffering from fatigue, joint, and muscle pain. This finding was supported by a finding of study conducted in Nepal, where malaria and dengue co-infections were associated with fatigue, joint pain, and chills [30]. This finding will facilitate early diagnosis and appropriate treatment of patients co-infected with malaria and dengue.

In this study, the prevalence of malaria and dengue co-infections among febrile patients was estimated for the first time in Kassala, eastern Sudan. Malaria and dengue co-infections are considered more severe than mono- infection, such as in a study conducted in French Guiana [25]. Malaria and dengue co-infections can cause severe clinical conditions such as increased platelet and haemoglobin levels [25]. Malaria and dengue co-infections exist basically among the population living in endemic areas with both malaria and dengue [31]. Malaria and dengue infections usually show asymptomatic or non-specific symptoms such as fever, headache, fatigue and malaise [32]. Both malaria and dengue have direct and indirect implications on the health system. A study conducted in Sri Lanka found that dengue requiring hospitalization had a substantial economic burden on the health system in the country [33]. Moreover, malaria infection has a substantial direct and indirect cost particularly in developing countries [34].

One of the limitations of the current study is that although the study is based on health facilities the clinical information is incomplete, because of deficiencies in the resources in the participating facilities. Some basic laboratory data could have been collected to characterize this condition and enable comparison with published reports on this condition, like malaria parasite count, haemoglobin concentration, platelet count and white blood cell count. Additionally, in the cross-sectional study design, causal link between the outcome and the exposure cannot be established.

## Conclusions

The prevalence of malaria and dengue co-infections was estimated in Kassala, eastern Sudan. The severity of clinical symptoms of patients with malaria and dengue co-infections was observed, and young ages were more affected by the co-infections. A clear understanding of the epidemiology of malaria and dengue co-infections is essential to inform decision-makers to institute an appropriate control strategy for both diseases. More research on malaria and dengue co-infections is needed in other endemic areas of Sudan. In addition, more vector studies are required for a better understanding of their role in transmission of malaria and dengue in Kassala, and in the country as a whole.

## Supporting information

S1 File Results of the distribution of malaria and dengue co-infections among age groups in Kassala state, eastern Sudan. (DOCX)

S2 File Results of the distribution of malaria and dengue co-infections among gender in Kassala state, eastern Sudan. (DOCX)
