# Supplementary material for: Prevalence of malaria and dengue co-infections among febrile patients during dengue transmission season in Kassala, eastern Sudan
Source: PLoS Negl Trop Dis. 2023 Oct 4;17(10):e0011660. doi: 10.1371/journal.pntd.0011660 (PMC10550125; doi:10.1371/journal.pntd.0011660)
Supplement: S1 File — (DOCX) [file pntd.0011660.s001.docx]

**S1 File. Results of the distribution of malaria and dengue co-infections among age groups**

**in Kassala state, eastern Sudan.**
